# Emotional response to amyloid beta status disclosure among research participants at high dementia risk

**DOI:** 10.1002/alz.70115

**Published:** 2025-05-07

**Authors:** Sapir Golan Shekhtman, Michal Schnaider Beeri, Ramit Ravona Springer, Maya Zadok, Mery Ben Meir, Yael Rosen‐Lang, Revital Shutsberg, Dar Gelblum, Tal Niv, Adar Matatov, Anthony Heymann, Joseph Azuri, Ithamar Ganmore, Chen Hoffman, Liran Domachevsky, Orit H. Lesman‐Segev

**Affiliations:** ^1^ The Joseph Sagol Neuroscience Center Sheba Medical Center Ramat Gan Israel; ^2^ Faculty of Medicine and Health Sciences Tel Aviv University Tel Aviv Israel; ^3^ Krieger Klein Alzheimer's Research Center Brain Health Institute Piscataway New Jersey USA; ^4^ Memory Clinic Sheba Medical Center Ramat Gan Israel; ^5^ Department of Family Medicine Maccabi Health Services Tel Aviv Israel; ^6^ Neurology Department Sheba Medical Center Ramat Gan Israel; ^7^ Department of Diagnostic Imaging Sheba Medical Center Ramat Gan Israel

**Keywords:** Alzheimer's disease, amyloid beta, amyloid beta positron emission tomography imaging, amyloid beta status disclosure, anxiety, cognitively normal individuals, depression, emotional responses, motivation for risk‐reduction behavior, observational trials, subjective memory complaints

## Abstract

**INTRODUCTION:**

Amyloid beta (Aβ) can be detected in vivo years before Alzheimer's disease (AD) symptom onset and, according to recent criteria, is sufficient for a biological diagnosis. This study evaluates emotional responses to Aβ status disclosure in cognitively normal individuals.

**METHODS:**

Questionnaires were given before and 6 months after Aβ positron emission tomography results disclosure to assess anxiety and depression related to the possibility of an elevated result, subjective memory complaints, and motivation for risk‐reduction behavior.

**RESULTS:**

One hundred ninety‐nine cognitively normal adults were included. Non‐elevated Aβ status disclosure was associated with reductions in all emotional parameters compared to baseline (*p* < 0.001). Elevated Aβ disclosure was associated with no changes in depression or memory complaints and a modest decrease in anxiety and motivation to change lifestyle (*p* < 0.048).

**DISCUSSION:**

No negative psychological effects were observed after Aβ status disclosure. Decreased motivation for lifestyle changes was seen after disclosure of both elevated and non‐elevated amyloid status and should be targeted.

**Highlights:**

- No negative psychological effects were observed after amyloid beta (Aβ) status disclosure.
- Motivation for lifestyle changes decreased after Aβ disclosure.
- Lower education may predict “unfavorable” response to non‐elevated Aβ disclosure.
- Results support personalized communication strategies for Aβ disclosure.

## 1 BACKGROUND

Alzheimer's disease (AD) biomarkers are increasingly important as measures of the presence and progression of AD pathophysiology to supplement clinical diagnosis. With the recent approval of disease‐modifying AD treatments, AD biomarkers are required for treatment eligibility and may be used as markers for treatment success. Available AD biomarkers include amyloid beta (Aβ) and phosphorylated tau, the defining features of AD pathology.

Aβ deposition starts early during AD pathophysiology. It can be accurately and reliably detected in vivo, via fluids (cerebrospinal fluid [CSF] and, more recently, blood) or using positron emission tomography (PET) brain imaging, years and even decades before symptom onset.^1^ As a group, cognitively normal individuals with abnormal Aβ biomarkers have a more rapid progression of atrophy, hypometabolism, and cognitive decline.^2^ However, Aβ deposition occurs in high percentages of cognitively normal older adults (≈ 10% in age 60–70 years and > 50% in age 80–90 years), and even more so in apolipoprotein E (*APOE*) ε4 carriers. It can also accompany other primary neurodegenerative pathologies, specifically dementia with Lewy bodies and cerebral Aβ angiopathy.^3^ Recent National Institute on Aging–Alzheimer's Association revised criteria for diagnosis and staging of AD consider Aβ deposition as sufficient to establish the diagnosis of AD.^2^, ^4^ The absence of biomarker support of Aβ deposition can reliably rule out the presence of current Aβ deposition and, thus, current AD. However, it cannot foresee the development of AD in the future. This complex clinical interpretation and the potential that patients will make inaccurate assumptions about risk and future outcomes based on Aβ status is the reason that appropriate use criteria for Aβ biomarker testing currently recommend against testing and disclosing Aβ status in asymptomatic individuals.^5^, ^6^ That said, the availability of proven preventative therapies would likely alter this judgment.^5^ The recent approval of Aβ‐lowering drugs for the treatment of mild cognitive impairment (MCI) and early dementia due to AD is a step forward toward preventative therapies. When such treatments exist, cognitively normal adults will likely be screened with Aβ biomarkers to identify individuals with asymptomatic AD for targeted therapies. Thus, there is a need to better understand the emotional reaction of cognitively normal adults to the disclosure of Aβ status to fully understand the potential benefits and harms of Aβ result disclosure and to develop evidence‐based guidelines for its use in clinical practice.

In this study, we aimed to assess the emotional reaction to Aβ PET result disclosure in terms of anxiety and depression related to the possibility of an elevated Aβ result, subjective memory complaints (SMCs), and the willingness to perform lifestyle changes in cognitively normal adults at high AD risk, participants of observational or interventional studies. We further aimed to characterize the factors that put an individual at risk for an “undesired” response to disclosure, which may be used to facilitate a more “personalized disclosure.”

## 2 METHODS

### 2.1 Participants

Cognitively normal adults at risk for AD and related dementia (ADRD) were recruited from two observational studies (the Israel Registry for Alzheimer's Prevention [IRAP]^7^ and the Israel Diabetes and Cognitive Decline [IDCD] study^8^) and two interventional trials (the Human Application Combined Approach for Prevention of Alzheimer's Disease [HAPPCAP‐AD; NCT05256121] and the Cognitive‐Motor Intervention Using Virtual Reality for Middle‐Aged Individuals at High Dementia Risk [VRAP;^9^ NCT02832921]) that incorporate Aβ PET scans. Inclusion criteria for all studies require that participants are cognitively normal at baseline; without a history of severe neurological, psychiatric, or other diseases that might affect cognition; and fluent in Hebrew. IRAP, HAPPCAP‐AD, and VRAP participants are offspring of patients with AD who are between the ages of 40 and 65 years at recruitment.^7^ HAPPCAP‐AD additionally requires the presence of at least two ADRD‐risk factors (diabetes, obesity, hypertension, sedentary lifestyle, smoking, and depression). IDCD participants are ≥ 65, have type 2 diabetes (T2D), have a reliable informant, are independent, and reside in the greater Tel Aviv area. Participants in all studies are insured by Maccabi Health Services, Israel's second‐largest health maintenance organization (HMO), providing detailed medical information on each participant since 1998. Exclusion criteria for Aβ PET scans include depression (Geriatric Depression Scale > 5) or anxiety (Beck Anxiety Inventory > 35) symptoms in the year preceding the scan.

Written informed consent was obtained from all participants. Participants received verbal and written standardized information regarding Aβ brain deposition, the scan procedure, the implication of a positive (an elevated Aβ deposition) and a negative (a non‐elevated Aβ deposition) scan result, and the questionnaires that they will be requested to fill out before the scan and 4 to 6 months after it. The study was conducted under the institutional review board approval of Sheba Medical Center and Maccabi Health Services.

### 2.2 Questionnaires

Pre‐scan questionnaires were completed during the scan day, before the scan. A similar follow‐up questionnaire was given to the participant in a separate phone call ≈ 6 months after disclosure. Both pre‐PET and post‐PET questionnaires consisted of semi‐quantitative questions on a scale of one to five (1–non; 2–mild; 3–moderate; 4–severe; 5–very severe) that assess the emotional reaction to Aβ PET result disclosure in four main domains: (1) anxiety related to the possibility of an elevated result—before the scan: “What is your level of anxiety from the possibility of a positive scan result?” / after disclosure: “What is your level of anxiety after the result disclosure?”; (2) depression related to the possibility of an elevated result—before the scan: “What is your level of depression regarding the possibility of elevated Aβ levels?” / after disclosure: “What is your level of depression after the result disclosure?”; (3) SMCs—before the scan: “Do you experience a decline in your memory (not including forgetting the location of items, dates, or names)?” / after disclosure: “Do you experience a decline in your memory since the scan result disclosure (not including forgetting the location of items, dates, or names)?”; (4) motivation to conduct changes in risk‐reduction behaviors—before the scan: “How motivated are you to make lifestyle changes (such as physical activity and nutritional habits) based on the scan result?” / after disclosure: “How ready/motivated are you to make lifestyle changes based on the scan result?”

### 2.3 Aβ PET scan acquisition, processing, visual read, and interpretation

#### 2.3.1 Acquisition

Aβ PET scans were performed at the Sheba Medical Center nuclear medicine department on a Philips Vereos PET/computed tomography (CT) scanner in 3D acquisition mode. A low‐dose CT scan was performed for attenuation correction before all scans. The acquisition began 90 minutes post‐injection of 4 to 5 millicuries of [F18] Flutemetamol Vizamyl TM (GE Healthcare) and took 20 minutes (https://www.accessdata.fda.gov/drugsatfda_docs/label/2017/203137s008lbl.pdf).

#### 2.3.2 Processing and quantification

PET images were co‐registered to the participants’ T1 structural magnetic resonance images using Statistical Parametric Mapping 12 (Wellcome Department of Imaging Neuroscience, Institute of Neurology). FreeSurfer 7.1 was used for parcellation, and standardized uptake value ratio (SUVR) maps were created using the whole cerebellum as a reference region. Global SUVR values were extracted from a large cortical region of interest that included frontal, parietal, cingulate, and temporal cortices. A global SUVR score of 1.21 was the threshold used for an elevated Aβ PET scan result.^10^

#### 2.3.3 Visual reads

Images were read in agreement by a trained neuroradiologist and a nuclear medicine physician blinded to quantification results. Both had completed the electronic training program (https://www.readvizamyl.com/) developed by GE Healthcare for the interpretation of F18‐Flutemetamol images.

**RESEARCH IN CONTEXT**

1. **Systematic review** The literature was reviewed using traditional sources (e.g., PubMed). Prior studies suggest amyloid beta (Aβ) status disclosure carries low psychological harm, but most studies are limited by small cohorts or individuals screened for interventional trials, limiting generalizability. Additionally, the impact on motivation for lifestyle changes and predictors of “unfavorable” emotional responses remain underexplored.
2. **Interpretation** Emotional response to Aβ status disclosure was assessed in a large cohort of cognitively normal adults, predominantly from observational trials. We observed no significant harm after disclosure. However, motivation to adopt lifestyle changes decreased after disclosing both elevated and non‐elevated Aβ status. Lower education was identified as a possible predictor of “unfavorable” emotional response to non‐elevated Aβ disclosure.
3. **Future directions** Future research should focus on identifying populations with elevated psychological vulnerability and developing personalized communication strategies. Mechanisms behind decreased motivation for lifestyle changes after disclosure should be further explored and targeted.

#### 2.3.4 Interpretation

Images were interpreted by combined visual read and quantification in agreement. In cases of disagreement between the visual read and quantification result, a third experienced PET reader gave a final tiebreaker read, blinded to clinical information, quantification, and the first visual read.

### 2.4 Aβ PET status disclosure

PET result disclosure was performed verbally in a structured phone call followed by a letter sent to the participant through the mail. A research coordinator disclosed non‐elevated Aβ PET scan results. The disclosure included a discussion about the meaning of a non‐elevated Aβ level, emphasizing that although there was no evidence of medium‐to‐high Aβ deposition in the brain, there is no guarantee against future deposition. Elevated Aβ PET scan results were disclosed by the study's principal investigator in a phone call that included a discussion about the meaning of the findings, taking the participant's age into account (high predictive value for cognitive decline due to AD at younger age and low predictive value for cognitive decline due to AD at older age). The concept of brain amyloidosis as a risk factor for future cognitive decline was addressed, and recommendations were made to treat cardiovascular risk factors if they exist; eat a balanced Mediterranean diet; and keep a physically, cognitively, and socially active lifestyle. Neurological and neurocognitive assessments were recommended if not done in the past year or if the participant reported any subjective concerns. Otherwise, routine follow‐ups were recommended based on the original study protocol. Patients were encouraged to contact the study coordinator with any questions, concerns, or changes in cognition.[Fig alz70115-fig-0001]

### 2.5 Statistical analysis

Participants who received non‐elevated and elevated Aβ PET scan results were compared across demographic and clinical characteristics and baseline responses using the independent‐samples Mann–Whitney *U* test. Non‐dropouts and dropouts were compared across demographic and clinical characteristics using a Wilcoxon signed‐rank test for continuous variables. Within‐group changes after PET result disclosure were tested using a paired Wilcoxon signed‐rank test, comparing pre‐scan and post‐result disclosure responses. The association between participants’ responses to a non‐elevated or an elevated Aβ PET scan result was assessed using mixed effects models. A model was fitted for each of the following response variables: anxiety and depression related to the possibility of an elevated result, SMCs, or motivation to change lifestyle. The models included fixed effects (timepoint [pre/post‐PET], result [non‐elevated/elevated], and timepoint*result) and random effects (age, sex, education, baseline Mini‐Mental State Examination [MMSE]). As a secondary analysis, the Mann–Whitney test and Fisher exact test were used to identify possible predictors of an “unfavorable” response to a non‐elevated Aβ status disclosure, defined as an increase in anxiety/depression/SMCs/motivation to conduct changes in risk‐reduction behaviors. Statistical significance was defined as *p* < 0.05. Statistical analysis was performed using IBM SPSS Statistics for Windows, Version 24.0 (IBM Corp.). Diagrams were created using SankeyMATIC.

## 3 RESULTS

### 3.1 Cohort characteristics

**FIGURE 1 alz70115-fig-0001:**
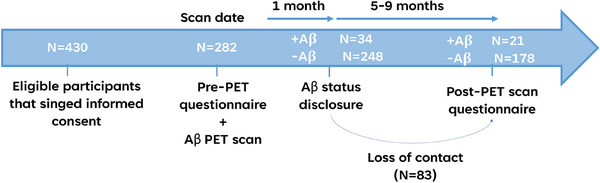
Study design. +Aβ, participants with elevated Aβ levels; −Aβ, participants with non‐elevated Aβ levels. Aβ, amyloid beta; PET, positron emission tomography.

Four hundred fifty‐seven participants were approached, provided with information about Aβ PET scans, and signed informed consent; 282 individuals completed the pre‐PET questionnaire and underwent an Aβ PET scan between 2017 and 2022. One hundred ninety‐nine participants completed the 6‐month post‐PET questionnaire and were included in this study (Figure 1); the other 83 participants were not included due to loss of contact or unwillingness to participate in the post‐PET phone questionnaire. Participants who dropped out of the study were older (median 68.9 years [60.9–78.4] vs. median 65.2 years [64–73.5]; *p* = 0.008) and had slightly lower MMSE scores (median 29 [28–30] for both groups, 56.7% of the dropout vs. 70.7% of the study sample had MMSE ≥ 29; *p* = 0.023), but otherwise not different from the study group (Table S1 in supporting information).

Twenty‐one (10.6%) of the Aβ PET scans of the study cohort exhibited cortical uptake, indicating elevated Aβ levels. Participants with an elevated Aβ result were older (median 62.5 years [55.4–71] vs. median 80.8 years [72.9–82.5]; *p* < 0.001, Table 1) and had slightly, though not statistically significant, lower MMSE scores (median 29 [27.5–30] vs. median 29 [28–30]; *p* = 0.070) compared to their counterparts with a non‐elevated Aβ result. While the rate of *APOE* ε4 carriers was higher in the Aβ‐elevated group, the difference did not reach statistical significance (20.8% vs. 42.9%; *p* = 0.138).

**TABLE 1:** Demographic, clinical characteristics, and baseline response.

Characteristics	Non‐elevated Aβ result (*n* = 178)	Elevated Aβ result (*n* = 21)	*p*
Demographics and clinical characteristics			
Observational study (IDCD/IRAP), *n* (%)	153 (85.6%)	21 (100%)	0.067
Age, median (IQR)	62.5 (55.4–71)	80.8 (72.9–82.5)	< 0.001^a^
Females, *n* (%)	102 (57.3%)	8 (38.1%)	0.095
Years of education, median (IQR)	16 (14–18.3)	14 (12–17.5)	0.097
*APOE* ε4 carrier, *n* (%)	37 (20.8%)	9 (42.9%)	0.138
MMSE, median (IQR)	29 (28–30)	29 (27.5–30)	0.070
SUVR, median (IQR)	0.9 (0.9–1)	1.3 (1.2–1.5)	< 0.001^a^
Baseline response non/mild/moderate/severe/very severe/missing *n* (%)			
Subjective memory complaints	64 (36%)/48 (27%)/46 (25.8%)/12 (6.7%)/8 (4.5%)/0 (0%)	10 (47.6%)/7 (33.3%)/3 (14.3%)/1 (4.8%)/0 (0%)/0 (0%)	0.127
Depression related to the possibility of an elevated result	62 (34.8%)/41 (23%)/49 (27.5%)/19 (10.7%)/7 (3.9%)/0 (0%)	13 (61.9%)/3 (14.3%)/2 (9.5%)/2 (9.5%)/1 (4.8%)/0 (0%)	0.051
Anxiety related to the possibility of an elevated result	57 (32%)/31 (17.4%)/42 (23.6%)/35 (19.7%)/13 (7.3%)/0 (0%)	9 (42.9%)/4 (19%)/3 (19%)/3 (14.3%)/1 (4.8%)/0 (0%)	0.260
Motivation to change lifestyle	11 (6.2%)/0 (0%)/22 (12.4%)/45 (25.3%)/99 (56.2%)/0 (0%)	2 (9.5%)/0 (0%)/2 (9.5%)/2 (9.5%)/15 (71.4%)/0 (0%)	0.329

*Note*: Comparison of demographics, clinical characteristics, and baseline response between non‐elevated and elevated Aβ groups (Mann–Whitney *U* test).

Abbreviations: Aβ, amyloid beta; *APOE*, apolipoprotein E; IDCD, Israel Diabetes and Cognitive Decline; IQR, interquartile range; MMSE, Mini‐Mental State Examination; IRAP, Israel Registry for Alzheimer's Prevention; SUVR, standardized uptake value ratio.

^a^ *p* < 0.001.

Comparing baseline (pre‐PET) questionnaire responses between the Aβ elevated and non‐elevated, no differences were found in SMCs and anxiety related to the possibility of elevated Aβ deposition and in motivation to make lifestyle changes. However, a trend‐level difference was found in depression related to the possibility of an elevated Aβ deposition (*p* = 0.051), in which the non‐elevated group had higher depression levels compared to the Aβ‐elevated group.

### 3.2 Participants’ response to Aβ status disclosure

#### 3.2.1 SMCs

**FIGURE 2:**
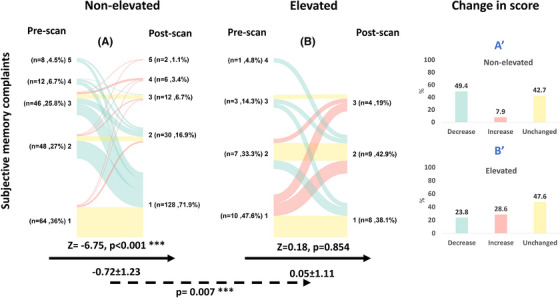
SMCs before and after the disclosure of a non‐elevated or an elevated Aβ result. A colored line connects each subject's SMCs score (1–lowest SMCs, 5–highest SMCs) before and after disclosure. The color indicates whether there has been a decrease (green), increase (red), or no change (yellow) in the response. The black arrow represents the within‐group comparison between the pre‐ and post‐PET responses (Wilcoxon signed‐rank test), while the absolute within‐group change in score after disclosure is presented beneath it (value, SD). The dashed arrow represents the between‐group comparison of the change in response between the non‐elevated (A) versus the elevated (B) Aβ groups (mixed effects models; *p* value of the timepoint x result is presented). Bar plots describe the group‐level change of SMCs after the disclosure of non‐elevated Aβ result (A′) or elevated Aβ result (B′). *P* value. *p < 0.05, *p < 0.01, ***p < 0.001. Aβ, amyloid beta; PET, positron emission tomography; SD, standard deviation; SMCs, subjective memory complaint.

In the pre‐PET questionnaire, most participants reported no or mild SMCs in both the non‐elevated Aβ group (63%) and the elevated Aβ group (80.9%), with no significant difference between the two groups at baseline (*p* = 0.127, Table 1). After the disclosure of a non‐elevated Aβ result, 88 individuals (49.4%) reported lower SMCs on their post‐PET questionnaire (Figure 2A,A′), resulting in a significant reduction of the average score by 0.72 units (post‐PET—pre‐PET; *Z* = −6.75; *p* < 0.001). After the disclosure of an elevated Aβ result, 10 individuals (47.6%) reported a similar score, and 6 (28.6%) reported a higher score on their post‐PET questionnaire (Figure 2B,B′), resulting in a non‐significant increase of the average score by 0.05 units (post‐PET—pre‐PET; *Z* = −0.18; *p* = 0.854). The change in score after disclosure was different in the elevated Aβ group compared to the non‐elevated Aβ group (*p* = 0.007, Figure 1) such that individuals receiving a non‐elevated Aβ result had a more “comforting” response, that is, decreased SMCs score.

#### 3.2.2 Depression related to the possibility of an elevated result

**FIGURE 3:**
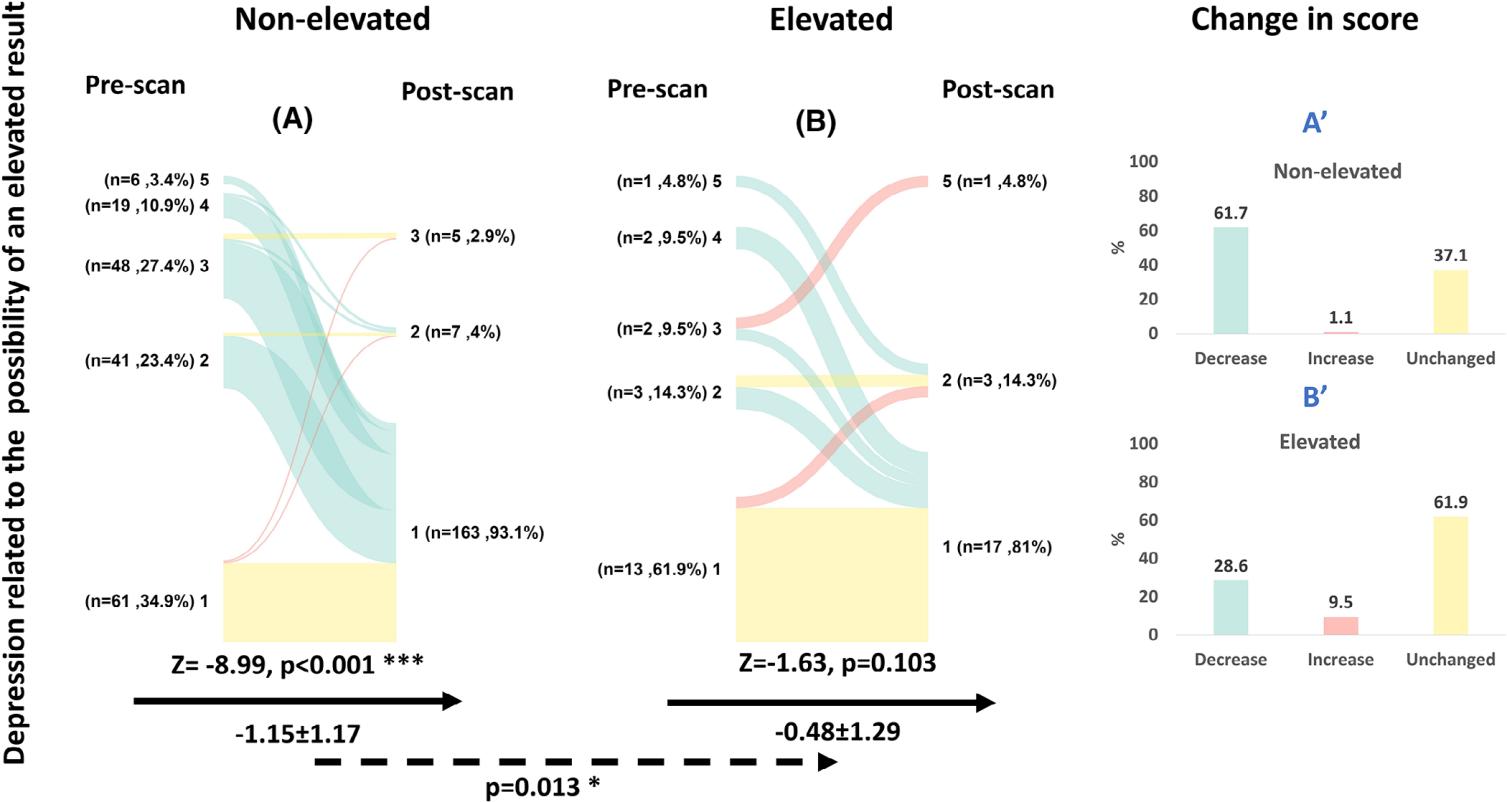
Depression related to the possibility of an elevated result before and after the disclosure of a non‐elevated or an elevated Aβ result. A colored line connects each subject's depression score (1–lowest depression, 5–highest depression) before and after disclosure. The color indicates whether there has been a decrease (green), increase (red), or no change (yellow) in the response. The black arrow represents the within‐group comparison between the pre‐ and post‐PET responses (Wilcoxon signed‐rank test), while the absolute within‐group change in score after disclosure is presented beneath it (value, SD). The dashed arrow represents the between‐group comparison of the change in response between the non‐elevated (A) versus the elevated (B) Aβ groups (mixed effects models; *p* value of the timepoint x result is presented). Bar plots describe the group‐level change in depression scores after the disclosure of a non‐elevated Aβ result (A′) or an elevated Aβ result (B′). *P* value. *p < 0.05, *p < 0.01, ***p < 0.001. Aβ, amyloid beta; PET, positron emission tomography; SD, standard deviation.

“No depression” was the most common response before the scan in both the group receiving a non‐elevated Aβ PET result (34.9%) and an elevated Aβ PET result (61.9%), with a trend difference in depression levels between groups at baseline (*p* = 0.051, Table 1). After the disclosure of a non‐elevated Aβ result, 110 individuals (61.71%) reported lower depression scores on their post‐PET questionnaire (Figure 3A,A′), resulting in a significant reduction of the average score by 1.15 units (post‐PET—pre‐PET; *Z* = −8.99; *p* < 0.001). After the disclosure of an elevated Aβ result, 13 individuals (61.9%) reported no change in depression score, and 6 individuals (28.6%) reported a decrease in depression score on their post‐PET questionnaire (Figure 3B,B′), resulting in a non‐significant reduction of the average score by 0.48 units (post‐PET—pre‐PET; *Z* = −1.63; *p* = 0.103). The difference in change in response between the two groups was significant (*p* = 0.013).

#### 3.2.3 Anxiety related to the possibility of an elevated result

**FIGURE 4:**
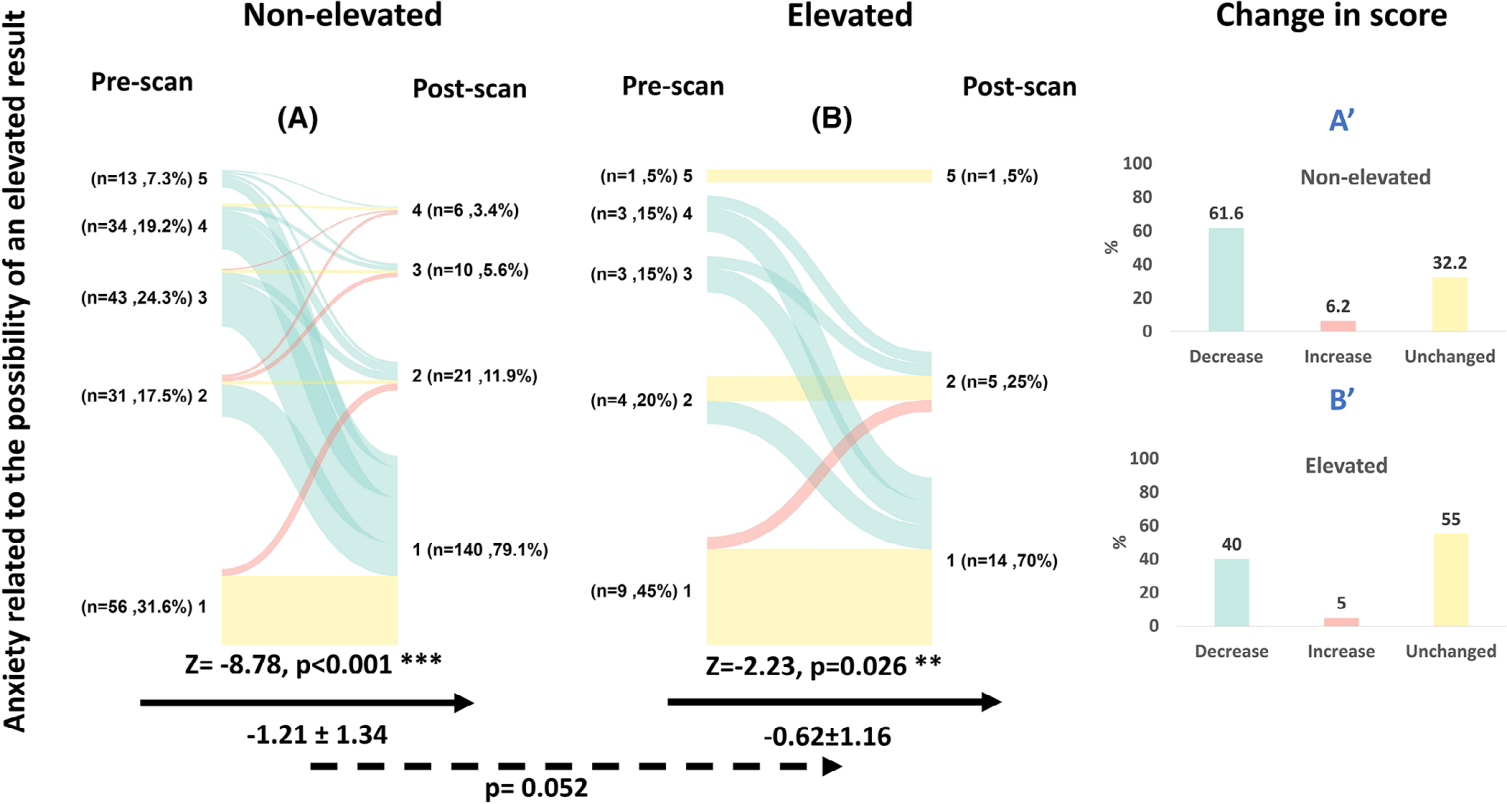
Anxiety related to the possibility of an elevated result before and after the disclosure of a non‐elevated or an elevated Aβ result. A colored line connects each subject's anxiety score (1–lowest anxiety, 5–highest anxiety) before and after disclosure. The color indicates whether there has been a decrease (green), increase (red), or no change (yellow) in the response. The black arrow represents the within‐group comparison between the pre‐ and post‐PET responses (Wilcoxon signed‐rank test), while the absolute within‐group change in score after disclosure is presented beneath it (value, SD). The dashed arrow represents the between‐group comparison of the change in response between the non‐elevated (A) versus the elevated (B) Aβ groups (mixed effects models; *p* value of the timepoint x result is presented). Bar plots describe the group‐level change in anxiety scores after the disclosure of a non‐elevated Aβ result (A′) or an elevated Aβ result (B′). *P* value. *p < 0.05, *p < 0.01, ***p < 0.001. Aβ, amyloid beta; PET, positron emission tomography; SD, standard deviation.

In both groups, “no anxiety” was the most common response in the pre‐PET questionnaire (31.6% in the non‐elevated Aβ group and 42.9% in the elevated Aβ group), with no significant difference in anxiety levels between the two groups at baseline (*p* = 0.260; Table 1). After the disclosure of a non‐elevated Aβ PET result, 109 individuals (61.6%) reported lower anxiety scores on their post‐PET compared to their pre‐PET questionnaires (Figure 4A,A′), resulting in a significant reduction of the average score by 1.21 units (post‐PET—pre‐PET; *Z* = −8.78; *p* < 0.001). After the disclosure of an elevated Aβ result, 8 individuals (40%) reported a lower score, and 11 (55%) reported a similar score of anxiety on their post‐PET questionnaire (Figure 4B,B′), resulting in a significant decrease of the average score by 0.62 units (post‐PET—pre‐PET; *Z* = −2.23; *p* = 0.026). The change in score after disclosure tended to be different between the group receiving a non‐elevated versus an elevated Aβ PET result, such that individuals receiving a non‐elevated Aβ result reported a greater decline in anxiety compared to the group with elevated Aβ (*p* = 0.052).

#### 3.2.4 Motivation to change lifestyle

**FIGURE 5:**
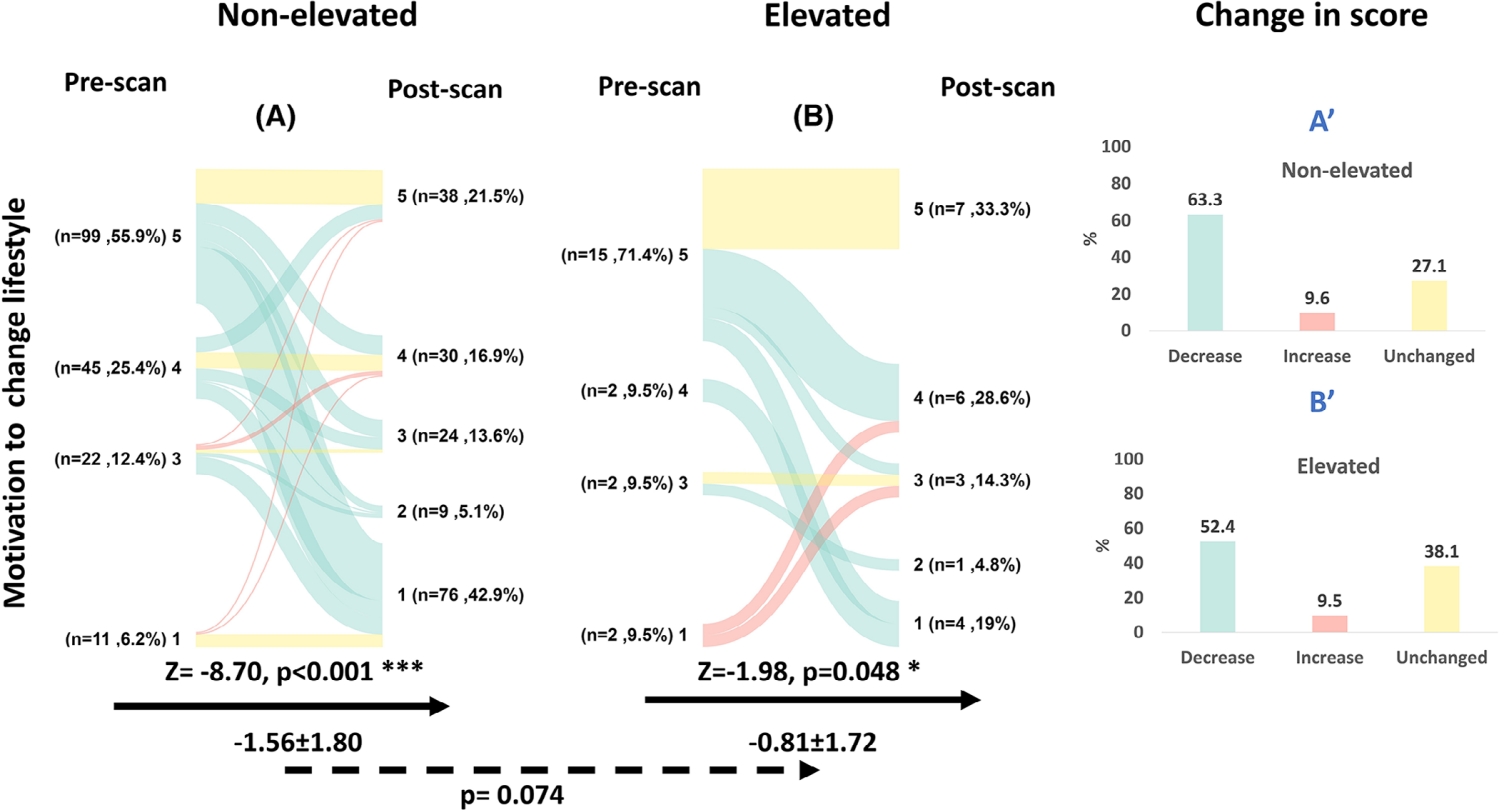
Motivation to change lifestyle before and after the disclosure of a non‐elevated or an elevated Aβ result. A colored line connects each subject's motivation to change lifestyle score (1–lowest motivation, 5–highest motivation) before and after disclosure. The color indicates whether there has been a decrease (green), increase (red), or no change (yellow) in the response. The black arrow represents the within‐group comparison between the pre‐ and post‐PET responses (Wilcoxon signed‐rank test), while the absolute within‐group change in score after disclosure is presented beneath it (value, SD). The dashed arrow represents the between‐group comparison of the change in response between the non‐elevated (A) versus the elevated (B) Aβ groups (mixed effects models; *p* value of the timepoint x result is presented). Bar plots describe the group‐level change in motivation to change lifestyle scores after the disclosure of a non‐elevated Aβ result (A′) or an elevated Aβ result (B′). *P* value. *p < 0.05, *p < 0.01, ***p < 0.001. Aβ, amyloid beta; PET, positron emission tomography; SD, standard deviation.

Most participants reported high or very high motivation to change their lifestyle in both the non‐elevated Aβ group (81.3%) and the elevated Aβ group (80.9%), with no significant difference between the groups at baseline (*p* = 0.329; Table 1). After the disclosure of a non‐elevated Aβ PET result, 112 individuals (63.3%) reported lower motivation to change their lifestyle on their post‐PET questionnaire (Figure 5A,A′), resulting in a significant reduction of the average score by 1.56 units (post‐PET—pre‐PET; *Z* = −8.70; *p* < 0.001). After the disclosure of an elevated Aβ result, 11 individuals (52.4%) reported lower motivation to change their lifestyle (Figure 5B,B′), resulting in a significant reduction of the average score by 0.81 units (post‐PET—pre‐PET; *Z* = −1.98; *p* = 0.048). The change in score after the disclosure of a non‐elevated Aβ result was not significantly different from an elevated result (*p* = 0.074).

### 3.3 Factors associated with an “unfavorable” response to a non‐elevated Aβ result disclosure

After receiving a non‐elevated Aβ result, most participants had an expected “favorable” response, that is, a decrease in anxiety related to the possibility of an elevated result/depression related to the possibility of an elevated result/SMCs/motivation to change lifestyle. In this case, a decrease in motivation to change lifestyle, though undesirable, was considered a favorable response because, under these circumstances, it likely reflects a calmer reaction to disclosure. Few participants, however, experienced an increase in these parameters despite the disclosure of a non‐elevated Aβ PET result. In a secondary analysis, we aimed to identify factors associated with these “unfavorable” responses.

Comparing the group with a non‐elevated Aβ status that had a “favorable” versus an “unfavorable” response, we found that persons who had an increase in SMCs had, on average, lower education (*p* = 0.022; Table 2), and persons who experienced an increase in anxiety related to the possibility of an elevated result were, on average, more likely to participate in an interventional study (*p* = 0.037; Table 2). Age, sex, and MMSE were not different in persons experiencing an unfavorable response (Table 2). Depression related to the possibility of an elevated result was not assessed because only four participants experienced an increase in depression levels after the result disclosure.

**TABLE 2:** Favorable versus unfavorable response to disclosure of a non‐elevated Aβ result.

	Subjective memory complaints			Anxiety related to the possibility of an elevated result			Motivation to change lifestyle		
Covariate	Favorable response *n* = 164	Un‐Favorable response *n* = 14	*p*	Favorable response *n* = 166	Un‐Favorable response *n* = 10	*p*	Favorable response *n* = 160	Un‐Favorable response *n* = 17	*p*
Type of study observational *n* (%)	141 (85.98%)	12 (85.71%)	0.615	145 (87.35%)	6 (60%)	**0.037** ^a^	138 (86.25%)	14 (82.35%)	0.442
Age median (IQR)	62.76 (55.58–70.73)	66.09 (54.11–79.26)	0.689	63.1 (55.54–71)	60.76 (53.71–64.80)	0.272	62.27 (55.39–70.87)	64.27 (57.76–77.69)	0.238
Sex females *n* (%)	92 (56.10%)	10 (71.43%)	0.204	94 (56.62%)	7 (70%)	0.313	93 (58.13%)	8 (47.10%)	0.267
Education median (IQR)	16 (15–18.75)	14 (12–16.75)	**0.022** ^a^	16 (14–18)	16.50 (16–19.5)	0.214	16 (14–18)	16 (14.5–20.5)	0.486
MMSE median (IQR)	29 (28–30)	29.00 (28.75–30)	0.507	29 (28–30)	29.50 (28.75–30)	0.580	29 (28–30)	29 (27.5–30)	0.251
*APOE* ε4 carrier *n* (%)^b^	36 (26.27%)	1 (0.73%)	0.191	36 (26.27%)	0 (0%)	0.206	36 (26.27%)	1 (0.73%)	0.321

*Note*: Mann–Whitney *U* test and Fisher exact test for comparison between participants who had a favorable response versus unfavorable response to a non‐elevated Aβ result.

Abbreviations: Aβ, amyloid beta; *APOE*, apolipoprotein E; IDCD, Israel Diabetes and Cognitive Decline; IQR, interquartile range; MMSE, Mini‐Mental State Examination.

^a^ Bold font indicates values with *p* < 0.05.

^b^ Data for *APOE* in only 137 participants with non‐elevated Aβ result.

## 4 DISCUSSION

We assessed the response of at risk, cognitively normal participants, mostly from observational studies, to Aβ status disclosure. We found that individuals receiving a non‐elevated Aβ status disclosure mostly show a “favorable response” with a decrease in memory complaints, anxiety, and depression, but also a decrease in motivation to perform risk‐reduction lifestyle changes. Individuals receiving an elevated Aβ status disclosure showed no change in SMCs or depression related to the possibility of an elevated result after disclosure. In contrast, their reported anxiety related to the possibility of an elevated result decreased, as did their motivation to perform lifestyle changes, though to a milder extent compared to individuals receiving a non‐elevated Aβ status disclosure. Finally, lower education and participation in an interventional trial were more likely in individuals who experienced an increase in SMCs or anxiety related to the possibility of an elevated result, respectively, despite a non‐elevated Aβ status disclosure.

Appropriate use criteria for Aβ biomarker testing currently recommend against testing and disclosing Aβ status in asymptomatic individuals.^5^, ^6^, ^11^ That said, Aβ PET was performed for research purposes under informed consent, and participants were given the option to receive the scan result. Only three participants chose not to receive it and subsequently declined to undergo the follow‐up questionnaires. All other participants requested disclosure of their Aβ status.

Our results are in agreement with previous studies that showed that the disclosure of Aβ status has a low risk for psychological harm both in elevated and non‐elevated Aβ levels. However, many of these studies assessed a small cohort or individuals screened for interventional trials, which may not accurately represent the total population of cognitively normal adults. Our work thus expands these findings to individuals participating in observational trials.

Participants receiving a non‐elevated Aβ PET result reported a statistically significant reduction in SMCs, anxiety, and depression, in line with previous studies^12^, ^13^ suggesting a feeling of relief and decreased worry when ruling out the presence of AD pathology. That said, several previous studies found no reduction in depression or anxiety after the disclosure of a non‐elevated Aβ status,^14^, ^15^, ^16^, ^17^ which may relate to a possible difference between participants in interventional trials versus observational trials and differences between cognitively normal participants with or without subjective cognitive decline (SCD) that were included in several of these studies.^14^, ^15^, ^16^, ^17^

Though most individuals experienced a “favorable” response after the disclosure of a non‐elevated Aβ result, a minority still experienced increased subjective memory decline, anxiety, or depression related to the possibility of an elevated result. Foreseeing a more personalized disclosure process, we assessed the factors that may put an individual at risk for such “undesired” responses. We found that lower education and participation in an interventional trial are more likely in individuals demonstrating these unfavorable responses. To the best of our knowledge, this is the first study that assesses the factors that facilitate safer disclosure of a non‐elevated Aβ status in individuals at high dementia risk. These findings align with results from the Amyloid Imaging to Prevent Alzheimer's Disease Diagnostic and Patient Management Study that showed that in individuals with SCD who had elevated Aβ levels, higher education was associated with lower distress‐associated disclosure.^7^ Our results indicate this may also be true in participants who are cognitively normal without SCD and have non‐elevated Aβ levels, suggesting a need for a more tailored disclosure process in these individuals. The limited number of participants with elevated Aβ levels in our cohort prevented analyses of “undesirable responses” in this group.

Twenty‐one individuals received a disclosure of an elevated Aβ result. During the disclosure process, a structured interview explained the meaning of the finding, the prevalence of Aβ positivity in the relevant age group, and the risk for cognitive decline in the future. The response to disclosure in this sub‐group was variable, with an average small decrease in anxiety related to the possibility of an elevated result and motivation to perform lifestyle changes and no difference in SMCs or depression associated with the possibility of an elevated result compared to the assessment before disclosure. Our cohort is too small to draw definitive conclusions. Still, the average decrease in anxiety related to the possibility of an elevated result and motivation to change lifestyle, though small, is counter‐intuitive considering the confirmation of the presence of one of the hallmark proteins of AD pathology. It may be related to the average old age of this sub‐cohort (median age 81 years), which was addressed during result disclosure as a relatively lower indicator for AD progression. These results are similar to those of others, which showed no effect of elevated Aβ result disclosure on depressive symptoms.^13^, ^14^, ^15^, ^16^, ^17^ Our results contrast with the findings reported in the Study of Knowledge and Reaction to Amyloid Testing (SOKRATES; *n* = 80, cognitively normal, 50 Aβ positives), in which participants who received an elevated Aβ result viewed the result as more serious and sensitive than other medical tests, and reported contemplating and making more changes to health behaviors and future plans.^12^ The average younger age of the SOKRATES Aβ positive cohort (70% were 64–75 years) and the fact that this cohort was screened for an interventional trial may explain the differences.

Both groups expressed strong motivation to implement lifestyle changes at baseline; however, after disclosure, a significant decrease in reported motivation was seen in both groups, though to a lesser extent in the elevated Aβ group. In the non‐elevated Aβ group, this may be attributed to the relief of not having AD pathology, allthough it still undesired. The causes for this decreased motivation are less understood in the elevated Aβ group. However, it is undesired, as keeping a healthy, active lifestyle is one of the current key recommendations for the prevention of AD.^18^, ^19^ This observation may be attributed to a potential false reassurance effect, underscoring the importance of maintaining a healthy lifestyle upon disclosure. To further explore this finding, we conducted additional analyses to identify potential characteristics associated with this phenomenon. A negative correlation was found between baseline motivation levels and the decline in motivation after the disclosure of a negative Aβ status (data not shown), likely due to the regression to the mean phenomenon. These findings highlight the need to ensure sustained engagement in lifestyle interventions. No demographic or other clinical factors were found to be significantly linked to the decline in motivation after disclosure. Future studies incorporating qualitative assessments and targeted questionnaires are needed to understand better the underlying factors related to this finding. In addition, more emphasis is necessary in addressing cardiovascular risk factors and promoting cognitive, social, and physically active lifestyles,^19^ regardless of the Aβ status, to ensure comprehensive health and well‐being in both groups.

Strengths of our study include the relatively large cohort of individuals with non‐elevated Aβ; the analyses that were focused on changes at the individual level rather than the group level, better capturing the variance in their distinct responses; and the fact that our cohort includes mainly participants from observational studies that may better represent the general population of cognitively normal adults compared to participants in interventional trials. That said, our cohort includes participants at high risk for ADRD, which still limits generalizability and may introduce selection bias. Our findings should also be interpreted within the following constraints of this study's limitations. Specifically, the absence of a control group consisting of participants who chose not to receive their Aβ status limits the ability to isolate external factors that may influence emotional change over time, regardless of Aβ status disclosure. In addition, the relatively small size of the Aβ‐elevated group (*n* = 21) limited our statistical power and the ability to analyze the factors associated with the reaction to disclosure in this population. Additionally, the study relied on semi‐quantitative self‐reported measures, introducing the potential for recall and social desirability biases. Individuals with pre‐existing depression and anxiety were excluded from the studies conducting amyloid PET for safety purposes, limiting the generalizability of the study and lowering the variance of baseline characteristics. Future research using larger, more diverse cohorts (in terms of risk factors, age, education, race, and ethnicity) that include a control group and more objective or detailed questionnaires could delve deeper into the impact of Aβ status disclosure on cognitive and psychological outcomes.

Foreseeing a future with preventative AD treatments, AD biomarkers are likely to play a vital role in screening cognitively normal adults to identify individuals with preclinical AD who are appropriate for treatment. Our findings suggest no psychological harm in elevated and non‐elevated Aβ PET scan status disclosure, strengthening findings from previous studies. We also show that individual variability exists, specifically in lower education, suggesting the need for a more tailored approach in this population. Finally, the decrease in motivation to implement lifestyle changes after the disclosure of elevated or non‐elevated Aβ status warns against false reassurance during the disclosure process.

## AUTHOR CONTRIBUTIONS

Sapir Golan Shekhtman, Michal Schnaider Beeri, Ramit Ravona Springer, and Orit H. Lesman‐Segev designed the study. Sapir Golan Shekhtman, Maya Zadok, Mery Ben Meir, Revital Shutsberg, Dar Gelblum, Tal Niv, Adar Matatov, Anthony Heymann, Joseph Azuri, Ithamar Ganmore, Chen Hoffman, and Liran Domachevsky helped with collecting the data. Yael Rosen‐Lang advised about the statistical analysis. Sapir Golan Shekhtman analyzed the data. Sapir Golan Shekhtman and Orit H. Lesman‐Segev interpreted the data and wrote the manuscript. All authors reviewed the manuscript. Orit H.Lesman‐Segev is the guarantor of this work and, as such, has full access to all the data in the study and takes responsibility for the integrity of the data and the accuracy of the data analysis.

## CONFLICT OF INTEREST STATEMENT

The authors have nothing to disclose. Author disclosures are available in the supporting information.

## CONSENT STATEMENT

All human subjects provided informed consent

## Supporting information



Supporting Information

ICMJE Disclosure Form

## Data Availability

The data collected for the current study are available from the corresponding author upon reasonable request.
