# Supplementary material for: Emotional response to amyloid beta status disclosure among research participants at high dementia risk
Source: Alzheimers Dement. 2025 May 7;21(5):e70115. doi: 10.1002/alz.70115 (PMC12056598; doi:10.1002/alz.70115)
Supplement: Supplementary file 1 — Supporting Information [file ALZ-21-e70115-s001.docx]

# Supplementary

| **Supplementary Table 1 – Demographic, clinical characteristics, dropouts vs non-dropouts** | | | |
| --- | --- | --- | --- |
| Characteristics | Dropouts (n=83) | Non-dropouts (n=199) | p |
| Observational study (IDCD/IRAP), n (%) | 77 (92.8%) | 174 (87.4%) | 0.193 |
| Age, Median (IQR) | 68.69 (60.9-78.4) | 63.99 (56.44-73.5) | 0.008** |
| Females, n (%) | 40 (48.2%) | 110 (55.3%) | 0.278 |
| Years of education, Median (IQR) | 16 (12-18) | 16 (14-18) | 0.150 |
| ApoE4 carrier, n (%) | 16 (23.5%) | 46 (29.1%) | 0.389 |
| MMSE, Median (IQR) | 29 (28-30) | 29 (28-30) | 0.023* |
| SUVR, Median (IQR) | 0.95 (0.9-1) | 0.94 (0.9-1) | 0.340 |
| Comparison of demographics and clinical characteristics (Independent-Samples Mann-Whitney U Test) Abbreviations: IQR, Interquartile Range; ApoE4, Apolipoprotein E4; MMSE, Mini-Mental State Examination; SUVR, Standardized uptake value ratio. *p<0.05, **p<0.01, *** p<0.001. | | | |
